# Challenges of rapid reviews for diagnostic test accuracy questions: a protocol for an international survey and expert consultation

**DOI:** 10.1186/s41512-019-0052-y

**Published:** 2019-04-04

**Authors:** Ingrid Arevalo-Rodriguez, Andrea C. Tricco, Karen R. Steingart, Barbara Nussbaumer-Streit, David Kaunelis, Pablo Alonso-Coello, Susan Baxter, Patrick M. Bossuyt, Javier Zamora

**Affiliations:** 1 Clinical Biostatistics Unit, Hospital Ramon y Cajal (IRYCIS), CIBER of Epidemiology and Public Health, Madrid, Spain; 2 Li Ka Shing Knowledge Institute, St. Michael’s Hospital. Epidemiology Division, Dalla Lana School of Public Health, University of Toronto, Toronto, Canada; 3 Queen’s Collaboration for Health Care Quality, Joanna Briggs Institute Centre of Excellence, Queen’s University, Kingston, Canada; 4 Cochrane Infectious Diseases Group, Liverpool School of Tropical Medicine, Liverpool, UK; 5 Cochrane Austria, Danube University Krems, Krems, Austria; 6 Canadian Agency for Drugs and Technologies in Health (CADTH), Ottawa, Canada; 7 Iberoamerican Cochrane Center-Servicio de Epidemiología Clínica y Salud Pública, Biomedical Research Institute (IIB-Sant Pau), Barcelona, Spain; 8 CIBER of Epidemiology and Public Health, Barcelona, Spain; 9 School of Health and Related Research (ScHARR), University of Sheffield, Sheffield, UK; 10 Department of Clinical Epidemiology & Biostatistics, Academic Medical Centre, University of Amsterdam, Amsterdam, The Netherlands

**Keywords:** Diagnostic tests, Accuracy, Rapid reviews, Knowledge synthesis

## Abstract

**Background:**

Assessment of diagnostic tests, broadly defined as any element that aids in the collection of additional information for further clarification of a patient’s health status, has increasingly become a critical issue in health policy and decision-making. Diagnostic evidence, including the accuracy of a medical test for a target condition, is commonly appraised using standard systematic review methodology. Owing to the considerable time and resources required to conduct these, rapid reviews have emerged as a pragmatic alternative by tailoring methods according to the decision maker’s circumstances. However, it is not known if streamlining methodological aspects has an impact on the validity of evidence synthesis. Furthermore, due to the particular nature and complexity of the appraisal of diagnostic accuracy, there is need for detailed guidance on how to conduct rapid reviews of diagnostic tests. In this study, we aim to identify the methods currently used by rapid review developers to synthesize evidence on diagnostic test accuracy, as well as to analyze potential shortcomings and challenges related to these methods.

**Methods:**

We will carry out a two-fold approach: (1) an international survey of professionals working in organizations that develop rapid reviews of diagnostic tests, in terms of the methods and resources used by these agencies when conducting rapid reviews, and (2) semi-structured interviews with senior-level individuals to further explore and validate the findings from the survey and to identify challenges in conducting rapid reviews. We will use STATA 15.0 for quantitative analyses and framework analysis for qualitative analyses. We will ensure protection of data during all stages.

**Discussion:**

The main result of this research will be a map of methods and resources currently used for conducting rapid reviews of diagnostic test accuracy, as well as methodological shortcomings and potential solutions in diagnostic knowledge synthesis that require further research.

## Introduction

Assessment of diagnostic tools, broadly defined as any element that aids in the collection of additional information for further clarification of the current status of a patient’s health [1], has emerged as a critical issue in health policy and global decision-making [2]. This rise has been mostly driven by the rapid technological developments of recent years, as well as a demand for the earlier identification of deleterious conditions, among other factors [2, 3]. Diagnostic evidence, including estimation of the accuracy of a test for detecting a target condition, in addition to comparing the accuracy of several tests, can be appraised using systematic reviews that follow rigorous methods [4–6]. In general, diagnostic accuracy studies focus on estimating the ability of the test(s) to correctly identify subjects with a predefined target condition, or the condition of interest (sensitivity) as well as to clearly identify those without the condition (specificity) [5].

Systematic reviews are currently the most widely used for searching, assessing and synthesising healthcare evidence [7]. However, the development of standard systematic reviews requires considerable time and resources, which may not be available in critical decision-making/time-sensitive scenarios [8, 9]. Recently, Beese et al. estimated that the probability of completing a Cochrane diagnostic test accuracy (DTA) review in 2 years was less than 10%, increasing to 33% in 4 years and to 58% in 8 years [10]. For intervention issues, rapid reviews have emerged as a pragmatic alternative to conventional systematic reviews by tailoring the methods according to the decision maker’s circumstances [4, 11–13]. Although a commonly accepted definition for rapid reviews does not yet exist in the literature, we define a rapid review as a knowledge synthesis strategy using limited or accelerated methods to expedite the time required to obtain a conclusive answer [11, 14]. Systematic review stages such as framing of the scope, literature searches and selection of eligible studies have been considered as key areas to improve the production time [14]. In addition, as a demand-driven product requested by end-users, rapid reviews are developed in a limited amount of time and with limited resources [15].

Despite the apparent advantages of rapid reviews, questions remain regarding the reliability of their conclusions in decision-making scenarios [16]. Recently, Marshall et al. assessed the impact of selected rapid review methods on the estimation of effect measures, concluding that rapid review approaches can be insufficient in scenarios where high precision is needed in the numerical estimation of the effect [17]. In addition, similar to conventional/aggregated data systematic reviews, there are some circumstances where rapid reviews can fail to provide answers to specific issues (such as subgroups of patients, or to analyze the influence of critical characteristics of the assessed test), and approaches such as individual-patient data analyses would be highly recommended [18–20].

An adequate level of comparative diagnostic accuracy is necessary to ensure a positive impact on final outcomes [21]. Due to the particular nature and complexity of the assessment of diagnostic accuracy, including the role and purpose of the test (e.g. screening or diagnosis), the setting of application, the identification/search for evidence, and the type of numerical information provided (i.e. sensitivity and specificity estimates), detailed guidance on how to conduct rapid reviews that address DTA questions is required. At present, few studies have addressed the impact of methodological shortcuts in diagnostic synthesis [22] and further information regarding the strategies currently in use is urgently needed [2, 23, 24].

This study belongs to a research series on the development of rapid reviews in diagnostic knowledge synthesis (further information is available at the Open Science Framework website: https://osf.io/uj7mc/?view_only=32189f1e47ce489482546a059a38a947). In this study, we will focus on identifying the current methods used by organizations that produce rapid reviews of diagnostic test. We will also explore the potential challenges and discuss the implications for further research.

## Methods

We will carry out a two-fold approach that includes an international survey of organizations developing diagnostic knowledge synthesis, and individual interviews with experts to identify shortcomings and challenges of the methodology used, as well as to suggest potential solutions for the future.

### International survey

We will carry out an international survey of current methods used by agencies that produce rapid reviews, based on a previous survey of rapid review developers [9]. In particular, we will contact current International Network of Agencies for Health Technology Assessment (INAHTA) members, the World Health Organization (WHO) collaborating centres on Health Technology Assessment (HTA), the Health Technology Assessment Network of the Americas (REDETSA) and the Health Technology Assessment International (HTAi) network non-profit members.

In order to obtain information on the methods and resources used by these agencies, we will develop an online questionnaire containing information about activities, methods and resources organized by review development stages [25]. The online survey will include the following issues:

1. General issues for the development of rapid reviews of diagnostic tests (including time for development, intended audience, commissioner of these reviews, focus of diagnostic reviews).
2. Composition of the review team for conducting of rapid reviews of diagnostic tests.
3. Methodological shortcuts used (including abbreviated search strategies, number of authors involved in the screening, selection of references and collection of data).
4. Methods for performing the synthesis of study results (including use of the GRADE system).
5. Issues regarding the preparation of the review report and their ending.

A pilot assessment of the survey will be performed by five external experts in conducting reviews and rapid reviews to ensure feasibility, duration and understandability. The final online survey, after feedback derived from the pilot assessment, will be conducted using SurveyMonkey (https://surveymonkey.com/). Participants will be invited via personalized emails, including a link to the online survey and a formal invitation letter. Regular reminders will be sent to increase the response rate.

After completion, data will be downloaded from the online platform to an Excel file and analyzed using STATA 15.0 (https://www.stata.com/). We will include all information retrieved, including incomplete surveys and we will adhere to CHERRIES guidance to report our survey findings [26].

### Semi-structured interview

Qualitative interviews will be used to further explore and validate the findings from the survey and to consider what is needed to address any potential challenges identified. We will use a purposeful sampling [27] to identify senior-level individuals and topic experts from relevant organizations in the field. We will seek individuals from different types of organizations and with different characteristics that may be important (e.g. years of experience, role) to ensure diversity of viewpoints. We expect that a sample of 10–12 individuals will give sufficient variation and enable data saturation to be achieved [28].

Semi-structured interviews will be carried out via GoToMeeting (https://www.gotomeeting.com/es-es). We will develop an interview guide that will take into consideration the results of the survey. Topics will include the following:

1. The validity of methods and resources employed by agencies and participants of the survey
2. The completeness of the essential list of methods and resources, reported by the survey participants
3. The challenges faced in conducting valid, accurate and useful rapid reviews of diagnostic tests in the future
4. Potential solutions to the critical challenges identified

A member of the research team will transcribe the interviews after data collection, and original recordings will be stored in a secure online data depository. During transcription, all identifying information will be removed to preserve anonymity and confidentiality of participants. A framework analysis approach will be used to examine the data. The initial framework will be based on the survey structure and results, with subsequent revisions and additions to develop a set of topics and sub-topics [29]. The initial analysis will be led by one researcher, with other members of the research team contributing to the development of the final framework. Atlas Ti software (https://atlasti.com/) will be used to aid systematic storage and retrieval of data during analysis, after which recordings and transcripts will be deleted.

### Ethical considerations and data protection

We will follow the Declaration of Helsinki ethical principles for the treatment of human participants, including ensuring informed consent prior to data collection, avoidance of coercion in recruitment, and preservation of confidentiality and anonymity [30]. Survey participants in the first element of the study will be given information about the study prior to commencing the survey and will be asked to acknowledge that they consent to take part. During the analysis, all identifying information will be removed to preserve anonymity and confidentiality of participants. Interview participants will be provided with an information sheet prior to taking part and asked to verbally agree to participate, which will be included in the recording. The interviews will take place at a convenient time for participants, and requests to stop recording for short periods during the interview will be allowed. All data relating to this study will be held in a secure repository for 5 years after the study has finished.

## Discussion

As the main result of this research, we will have an extensive map of methods and resources currently used for conducting DTA rapid reviews, as well as methodological shortcomings and potential solutions identified by experts in diagnostic knowledge synthesis, that require further research. We anticipate the following strengths and limitations of our study:

### Strengths

As the main strength of our research, we will combine two different methodologies to obtain a comprehensive overview of rapid review methods used for synthesis of DTA evidence. In addition, we will analyse the methodological challenges in this field, provided by experts in diagnostic evidence, in order to identify further research needed for the development of diagnostic evidence synthesis.

### Limitations

Currently, a commonly-accepted definition for rapid reviews does not exist. As such, there will be variation in the responses of participants in our study. To reduce the potential heterogeneity derived from this issue, we will provide a definition of rapid review in our study.

Likewise, our research might be limited to the rate of response of our survey by agencies producing DTA rapid reviews. We will send personalized emails to key individuals in each agency to ensure their participation. In addition, we will send regular reminders to increase the survey response rate. We will have an extended period to collect all potential responses from eligible participants. Analysis of survey findings will also depend on the profile of experts selected for semi-structured interviews. We will develop a list of critical characteristics in advance to ensure the diversity of viewpoints in this analysis.

Finally, we also expected that our findings will be applicable to diagnostic questions regarding the accuracy of a single test, as well as to the assessment of comparative accuracy of tests in a specific setting. However, special synthesis of test accuracy issues, such as incremental accuracy assessment, synthesis of predictive values or sensitivity only, are outside of the scope of our research.

### Further research

This study belongs to a research series on the development of rapid reviews in diagnostic knowledge synthesis. The findings of this study will be the initial step for further research on conducting DTA rapid reviews.

## References

[1] Knottnerus J, Frank B (2009). The evidence base of clinical diagnosis.

[2] Tricco AC, Langlois EV, Straus SE (2017). Rapid reviews to strengthen health policy and systems: a practical guide.

[3] Mustafa RA, Wiercioch W, Falavigna M, Zhang Y, Ivanova L, Arevalo-Rodriguez I, Cheung A, Prediger B, Ventresca M, Brozek J (2017). Decision making about healthcare-related tests and diagnostic test strategies. Paper 3: a systematic review shows limitations in most tools designed to assess quality and develop recommendations. J Clin Epidemiol.

[4] Moher D, Stewart L, Shekelle P (2015). All in the family: systematic reviews, rapid reviews, scoping reviews, realist reviews, and more. Syst Rev.

[5] Deeks J, Bossuyt P, Gatsonis CE (2010). Cochrane handbook for systematic reviews of diagnostic test accuracy.

[6] McInnes MDF, Moher D, Thombs BD, McGrath TA, Bossuyt PM, Clifford T, Cohen JF, Deeks JJ, Gatsonis C, Hooft L (2018). Preferred reporting items for a systematic review and meta-analysis of diagnostic test accuracy studies: the PRISMA-DTA statement. JAMA.

[7] Murad MH, Asi N, Alsawas M, Alahdab F (2016). New evidence pyramid. Evid Based Med.

[8] Ganann R, Ciliska D, Thomas H (2010). Expediting systematic reviews: methods and implications of rapid reviews. Implement Sci.

[9] Tricco AC, Antony J, Zarin W, Strifler L, Ghassemi M, Ivory J, Perrier L, Hutton B, Moher D, Straus SE (2015). A scoping review of rapid review methods. BMC Med.

[10] Beese S, Harris B, Davenport C, Mallet S, Takwoingi Y, Deeks JJ (2018). The first ten years of Cochrane DTA reviews: progress and common methodological challenges. Abstracts of the 25th Cochrane Colloquium: 2018.

[11] Hartling L, Guise JM, Kato E, Anderson J, Aronson N, Belinson S, Berliner E, Dryden D, Featherstone R, Foisy M (2015). EPC methods: an exploration of methods and context for the production of rapid reviews. Research white paper.

[12] Khangura S, Konnyu K, Cushman R, Grimshaw J, Moher D (2012). Evidence summaries: the evolution of a rapid review approach. Syst Rev.

[13] Polisena J, Garritty C, Kamel C, Stevens A, Abou-Setta AM (2015). Rapid review programs to support health care and policy decision making: a descriptive analysis of processes and methods. Syst Rev.

[14] Hartling L, Guise JM, Hempel S, Featherstone R, Mitchell MD, Motu’apuaka ML, Robinson KA, Schoelles K, Totten A, Whitlock E (2017). Fit for purpose: perspectives on rapid reviews from end-user interviews. Syst Rev.

[15] Tricco AC, Zarin W, Antony J, Hutton B, Moher D, Sherifali D, Straus SE (2016). An international survey and modified Delphi approach revealed numerous rapid review methods. J Clin Epidemiol.

[16] Nussbaumer-Streit B, Klerings I, Wagner G, Heise TL, Dobrescu AI, Armijo-Olivo S, Stratil JM, Persad E, Lhachimi SK, Van Noord MG (2018). Abbreviated literature searches were viable alternatives to comprehensive searches: a meta-epidemiological study. J Clin Epidemiol.

[17] Marshall I, Marshall R, Wallace B, Brassey J, Thomas J. Rapid reviews may produce different results to systematic reviews: a meta-epidemiological study. J Clin Epidemiol. 2018. PMID: 30590190 10.1016/j.jclinepi.2018.12.015. 10.1016/j.jclinepi.2018.12.015 PMC6524137 30590190

[18] Higgins JPT, Green S (editors). Cochrane Handbook for Systematic Reviews of Interventions Version 5.1.0 [updated March 2011]. The Cochrane Collaboration, 2011. Available from www.cochrane-handbook.org.

[19] Rogozinska E, Marlin N, Thangaratinam S, Khan KS, Zamora J (2017). Meta-analysis using individual participant data from randomised trials: opportunities and limitations created by access to raw data. Evid Based Med.

[20] Stewart LA, Tierney JF (2002). To IPD or not to IPD? Advantages and disadvantages of systematic reviews using individual patient data. Eval Health Prof.

[21] World Health Organization (2013). Health 2020: a European policy framework and strategy for the 21st century.

[22] van Enst WA, Scholten RJ, Whiting P, Zwinderman AH, Hooft L (2014). Meta-epidemiologic analysis indicates that MEDLINE searches are sufficient for diagnostic test accuracy systematic reviews. J Clin Epidemiol.

[23] Nussbaumer-Streit B, Klerings I, Wagner G, Titscher V, Gartlehner G (2016). Assessing the validity of abbreviated literature searches for rapid reviews: protocol of a non-inferiority and meta-epidemiologic study. Syst Rev.

[24] Haby MM, Chapman E, Clark R, Barreto J, Reveiz L, Lavis JN (2016). What are the best methodologies for rapid reviews of the research evidence for evidence-informed decision making in health policy and practice: a rapid review. Health Res Policy Syst.

[25] Pham B, Bagheri E, Rios P, Pourmasoumi A, Robson RC, Hwee J, Isaranuwatchai W, Darvesh N, Page MJ, Tricco AC (2018). Improving the conduct of systematic reviews: a process mining perspective. J Clin Epidemiol.

[26] Eysenbach G (2004). Improving the quality of web surveys: the Checklist for Reporting Results of Internet E-Surveys (CHERRIES). J Med Internet Res.

[27] Palinkas LA, Horwitz SM, Green CA, Wisdom JP, Duan N, Hoagwood K (2015). Purposeful sampling for qualitative data collection and analysis in mixed method implementation research. Admin Pol Ment Health.

[28] Miles MB, Huberman AM (1994). Qualitative data analysis: an expanded sourcebook.

[29] Gale NK, Heath G, Cameron E, Rashid S, Redwood S (2013). Using the framework method for the analysis of qualitative data in multi-disciplinary health research. BMC Med Res Methodol.

[30] World Medical Association (2013). World Medical Association Declaration of Helsinki: ethical principles for medical research involving human subjects. Jama.

